# Supplementary material for: Intake of Dietary Salicylates from Herbs and Spices among Adult Polish Omnivores and Vegans
Source: Nutrients. 2020 Sep 6;12(9):2727. doi: 10.3390/nu12092727 (PMC7551261; doi:10.3390/nu12092727)
Supplement: Supplementary file 1 [file nutrients-12-02727-s001.zip › Figure S1.pdf]

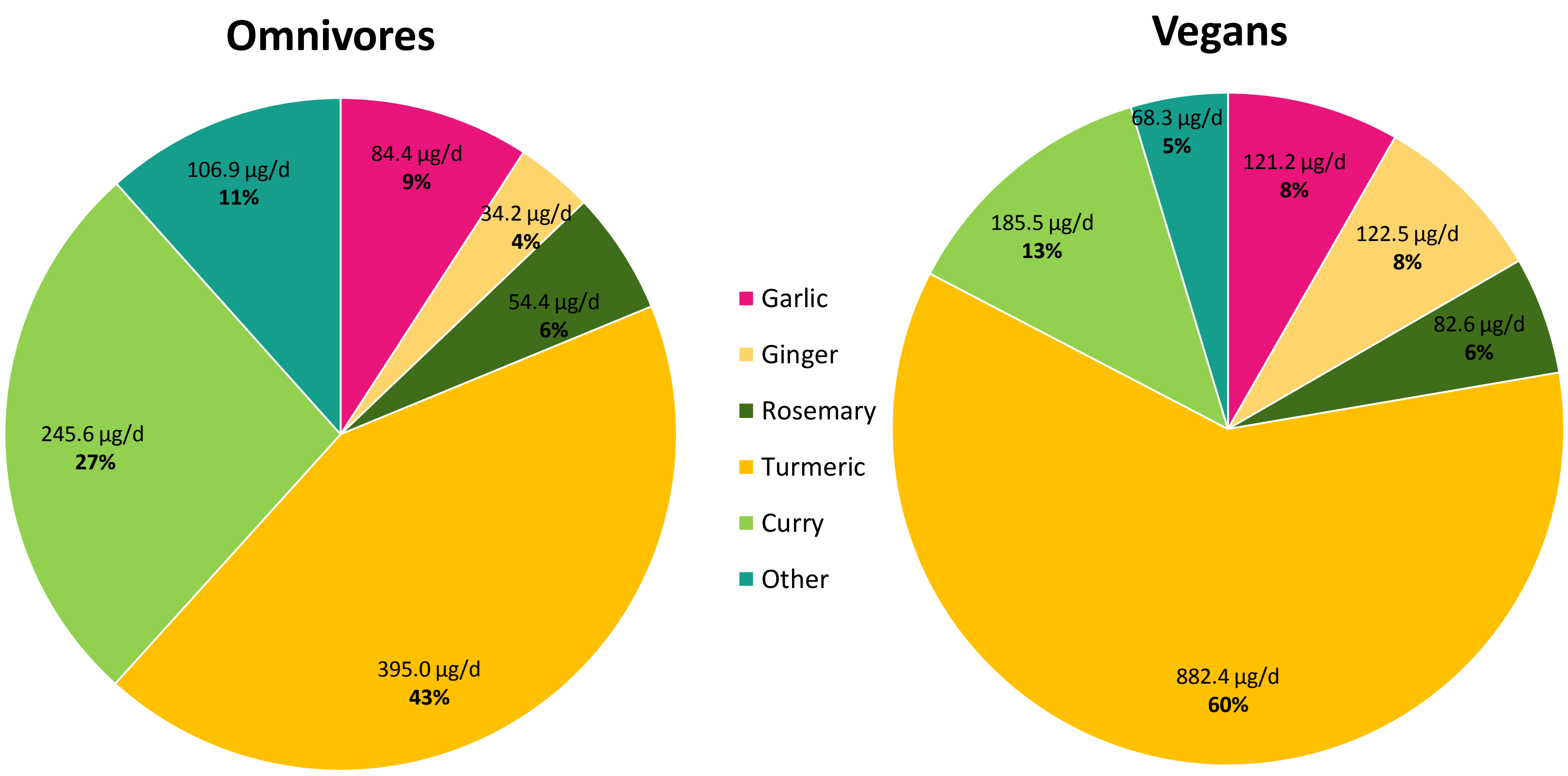

Fig. S1. The contribution of individual herbs and spices to the total dietary salicylates intake among adult omnivores and vegans
