# Supplementary material for: Intake of Dietary Salicylates from Herbs and Spices among Adult Polish Omnivores and Vegans
Source: Nutrients. 2020 Sep 6;12(9):2727. doi: 10.3390/nu12092727 (PMC7551261; doi:10.3390/nu12092727)
Supplement: Supplementary file 1 [file nutrients-12-02727-s001.zip › Table S1.pdf]

Table S1. Herbs and spices typical for vegan and mixed dishes

Meal	Vegan cuisine		Omnivorous cuisine	
	Sample of the dish	Herbs or spices	Sample of the dish	Herbs or spices
Breakfast	Porridge	vanilla, cocoa, cinnamon[s]	Porridge	vanilla, cocoa, cinnamon[s]
	Sandwiches with hummus	garlic, cumin[s], parsley[s]	Sandwiches with ham, cheese and tomato	pepper[s], basil[s], parsley[s]
	Pancakes with fruits	vanilla, turmeric[s]	Pancakes with cottage cheese	vanilla
	Tofu scramble	turmeric[s], garlic	Scrambled eggs	pepper[s], chive
	Vegetable pate	parsley[s], thyme[s], cumin[s], smoked paprika[s], ground ginger, nutmeg, pepper[s]	Traditional pate	bay leaves, allspice, pepper[s], nutmeg
Lunch	Vegetable soups with legumes	garlic, parsley[s], basil[s], thyme[s], dill, sweet pepper[s], hot pepper[s], black pepper[s], lovage	Broth with noodles	parsley[s], bay leaves, allspice, pepper[s]
	Seitan	soy sauce, oregano[s], thyme[s], garlic, sweet pepper[s],	Roasted meat eg. Roast beef	garlic, pepper[s], marjoram
	Spaghetti Napoletana	garlic, basil[s], parsley[s]	Spaghetti Bolognese	garlic, oregano[s], basil[s], pepper[s]
	Hindu dishes/ vegetable stew – tofu curry	garlic, ginger, curry[s], cayenne pepper[s], cilantro[s]	Traditional stew	sweet pepper[s], hot pepper[s], garlic, bay leaves, allspice, parsley[s]
Dinner	Tortilla with falafels	cumin[s], parsley[s], pepper, cilantro[s], cardamom, chili[s]	Sandwiches with eggs	pepper[s], dill
	Lentil dahl	garlic, pepper[s], garam masala[s], tandoori masala[s], turmeric, ginger[s], parsley[s]	Bigos (sauerkraut cabbage with meat)	pepper[s], bay leaf, allspice, caraway, marjoram
	Salad with cereals	garlic, parsley[s,] basil[s], dill	Salad caprese	pepper[s], basil[s]

[S] – herbs or spices rich in salicylates
